# Enhanced ROCK1 dependent contractility in fibroblast from chronic obstructive pulmonary disease patients

**DOI:** 10.1186/1479-5876-10-171

**Published:** 2012-08-22

**Authors:** Oskar Hallgren, Sara Rolandsson, Annika Andersson-Sjöland, Kristian Nihlberg, Elisabet Wieslander, Martina Kvist-Reimer, Magnus Dahlbäck, Leif Eriksson, Leif Bjermer, Jonas S Erjefält, Claes-Göran Löfdahl, Gunilla Westergren-Thorsson

**Affiliations:** 1 Department of Experimental Medical Science, BMC D12, Lund University, Lund, Sweden; 2 Department of Respiratory Medicine and Allergology, Lund University Hospital, Lund, Sweden; 3 AstraZeneca R&D, Lund, Sweden; 4 BMC D12, Klinikgatan 28, S-22184, Lund, Sweden

**Keywords:** Chronic obstructive pulmonary disease, Contractility, Emphysema, Fibroblast/myofibroblast, Rho-associated coiled-coil protein kinase 1

## Abstract

**Background:**

During wound healing processes fibroblasts account for wound closure by adopting a contractile phenotype. One disease manifestation of COPD is emphysema which is characterized by destruction of alveolar walls and our hypothesis is that fibroblasts in the COPD lungs differentiate into a more contractile phenotype as a response to the deteriorating environment.

**Methods:**

Bronchial (central) and parenchymal (distal) fibroblasts were isolated from lung explants from COPD patients (n = 9) (GOLD stage IV) and from biopsies from control subjects and from donor lungs (n = 12). Tissue-derived fibroblasts were assessed for expression of proteins involved in fibroblast contraction by western blotting whereas contraction capacity was measured in three-dimensional collagen gels.

**Results:**

The basal expression of rho-associated coiled-coil protein kinase 1 (ROCK1) was increased in both centrally and distally derived fibroblasts from COPD patients compared to fibroblasts from control subjects (p < 0.001) and (p < 0.01), respectively. Distally derived fibroblasts from COPD patients had increased contractile capacity compared to control fibroblasts (p < 0.01). The contraction was dependent on ROCK1 activity as the ROCK inhibitor Y27632 dose-dependently blocked contraction in fibroblasts from COPD patients. ROCK1-positive fibroblasts were also identified by immunohistochemistry in the alveolar parenchyma in lung tissue sections from COPD patients.

**Conclusions:**

Distally derived fibroblasts from COPD patients have an enhanced contractile phenotype that is dependent on ROCK1 activity. This feature may be of importance for the elastic dynamics of small airways and the parenchyma in late stages of COPD.

## Background

Chronic obstructive pulmonary disease (COPD) is characterized by a reduction in respiratory airflow that is not possible to fully normalize [1]. Several factors contribute to the reduced airflow. In central and small airways epithelial dysregulation results in impaired mucocilliary clearance, over-production of mucus, squamous cell metaplasia and subepithelial fibrosis [2,3]. Degradation of alveolar walls (emphysema), a hallmark of COPD, limits the area of gas exchange and reduces the elastic recoil. Cigarette smoke is undoubtedly the most contributing cause to these changes, as a majority of COPD patients have a history of heavy smoking. The inflammatory effect of cigarette smoke has been extensively examined and includes accumulation of inflammatory cells [2,4,5]. Inflammation per se can be regarded as part of a wound healing process. However, if the inflammatory stimuli persist, the process may become pathologic and mesenchymal cell phenotype alterations can be observed [6]. Fibroblasts are key players in wound healing as they are the main producer of extracellular matrix (ECM) and may adopt a contractile phenotype to promote wound closure [7,8]. A number of factors have been implicated in controlling fibroblast differentiation, including the growth factor TGF-β [9]. The compliance of the tissue is another determinant of the differentiation process [10]. The hallmark of a contractile fibroblast is a high expression of proteins involved in the contractile machinery which results in the formation of stress fibers [11]. The rho/ROCK pathway has been suggested to be a sensor of tissue compliance. As a result of increased tissue stiffness it may stimulate phosphorylation of myosin light chain kinase (MLCK) and subsequent assembly of stress fibers [11-13]. Fibroblasts contraction is also dependent on the expression of α-smooth muscle actin (α-SMA) [14]. Interestingly, it has been suggested that there exist different subsets of fibroblasts in central airways and in alveolar parenchyma, characterized by differences in morphology, production of ECM proteins and in the expression of α-SMA [15,16]. In a previous study we reported that these differences also included the production of proteoglycans [17]. In addition, the fibroblast phenotypes were altered in fibroblasts isolated from severe COPD patients.

The hypothesis of this study was that the altered phenotypes of centrally and distally derived fibroblasts from COPD patients also extend to their contractile properties. We especially wanted to investigate if the activity of rho/ROCK could account for such changes.

## Methods

### Patients

Patients (n = 9) suffering from very severe COPD (GOLD stage IV) who were undergoing lung transplantation at Lund University Hospital were included in the study [17]. The patients had stopped smoking at least 6 months before the lung transplantation. All patients were given a combination of different medicines. All of them were given glucocorticoids on regular basis. Control subjects were recruited from two different sources. First, non-smoking volunteers (n = 8) with no clinical history of any lung diseases were included in the study [18]. Written consent was obtained from all subjects. Second, lung explants from healthy donors (n = 4) with no history of lung disease were also included. Lungs were to be used for transplantation but could instead be included in this study as no matched recipients were available at that moment. In these cases written consent was obtained from their closest relatives. This study was approved by the Swedish Research Ethical Committee in Lund (FEK 91/2006, FEK 213/2005 and FEK 413/2008).

### Isolation of cells

Fibroblasts were isolated from explants from COPD patients as previously described [19]. Control fibroblasts were obtained from two different sources: from bronchial and transbronchial biopsies and explants from donors in situation when the lungs were not used for transplantation. Biopsies from control subjects were immediately after sampling transferred to cell culture medium (DMEM supplemented with 10% FBS, Gentamicin, PEST, and Amphotericin B (all from Gibco BRL, Paisley, UK)). After rinsing, bronchial and parenchymal pieces from biopsies were chopped into small pieces that were allowed to adhere to the plastic of cell culture flasks for 4 h and were then kept in cell culture medium in 37°C cell incubators until outgrowth of fibroblasts were observed. Bronchial and parenchymal fibroblasts were then referred to as centrally and distally derived fibroblasts, respectively.

Lung explants from COPD patients and donor lungs were dissected directly after removal from COPD patients and donors. Lung specimens were then immediately transferred to cell culture medium. Bronchial tissue was collected from the luminal side from the same localization as where bronchial biopsies were taken. Alveolar parenchymal specimens from explants were collected 2–3 cm from the pleura in the lower lobes, i.e. from the same location as where transbronchial biopsies were taken. Vessels and small airways were removed from the peripheral lung tissues. Bronchial and parenchymal specimens were chopped into small pieces and then treated as the biopsy material described above. All experiments were performed in passage 3–7. Within this range we could not see any correlation between passage number and any of the investigated parameters. The mean passage number when experiments were performed was 5.3 for centrally derived fibroblasts from control subjects, 4.6 for centrally derived fibroblasts from COPD patients, 5.3 for distally derived fibroblasts from control subjects and 5.3 for distally derived fibroblasts from COPD patients. The cell cultures were continuously stained with antibodies against vimentin and prolyl-4 hydroxylase to verify the mesenchymal identity and to estimate the purity. In the few cases when the cellular staining was less clear then the cell morphology was verified to be fibroblast-like and representative for the culture as a whole.

### Western blot

Cells were grown under standardized conditions and whole cell lysates were prepared using lysis buffer (50 mM Tris–HCl, 500 mM NaCl, 1% NP-40, 10% glycerol, 10 mM MgCl2 pH 7,4) containing the protein inhibitor complete mini (1 mM PMSF, 1 μg/ml Aprotinin, 1 μg/ml Pepstatin, 1 μg/ml Leopeptin, Roche, Manheim, Germany). Samples were solubilized in Laemmlis buffer and equal amounts of protein, 10 μg, were loaded and separated by electrophoresis on 4–12% Bis-Tris Gels (Invitrogen, Gibro, Carlsbad, CA). The proteins were blotted to PVDF membranes (Immobilon-P Transfer Membrane, Millipore Corporation, Billerica, MA). Membranes were incubated with antibodies against ROCK1 (Abcam, Cambridge, UK) , α-SMA (Abcam, Cambridge, UK), Rho-A (Santa Cruz Biotechnology, Inc. Santa Cruz, CA), and GAPDH (Santa Cruz Biotechnology, Inc. Santa Cruz, CA). Bound antibodies were visualized by Dy-light 700 and 800 nm conjugated secondary antibodies (Cell Signaling Technology Inc., Boston, MA). The fluorescence signal was detected on Odyssey® FC imaging system (LI-COR Biosciences, Lincoln, NE). Exposure times were standardized so that all samples were treated the same way for each antibody.

### Phalloidin staining

Fibroblasts (7000/well) grown overnight in 4-well chamber slides were fixed in 4% formaldehyde for 15 minutes and then permeabilized 0.1% Triton X for 10 minutes. Cells were then blocked in 2% BSA-TBS for 30 minutes and incubated with Alexa-fluor 488-conjugated phalloidin (Molecular Probes Invitrogen, Eugene, OR) for 30 minutes. Glasses were mounted using mounting media (Dako, Glostrup, Denmark). Cells were photographed using a TE2000-E fluorescence microscope (Nikon, Tokyo, Japan) equipped with a DXM1200C camera (Nikon).

### Immunohistochemistry

#### Immunostaining of fibroblasts

Fibroblasts (7000/well) grown overnight on chamber slides were fixed in 4% formaldehyde for 15 minutes and then permeabilized with 0.1% Triton X for 30 minutes. Cells were blocked in 2% BSA-TBS containing 5% goat serum (Vector laboratories, Burlingame, CA). Cells were incubated with primary antibodies: monoclonal mouse antibody against Prolyl 4-Hydroxylase (Acris antibodies, Hiddenhausen, Germany), monoclonal mouse IgM antibody against Vimentin (Santa Cruz Biotechnology, Santa Cruz, CA), polyclonal rabbit antibody against ROCK1 (Abcam, Cambridge, UK), monoclonal IgG and antibody against SM22-alpha (Abcam, Cambridge, UK), and with secondary antibodies: Alexafluor 488-conjugated goat anti-mouse antibody and Alexafluor 555-conjugated goat anti-mouse antibody (both from Molecular Probes Invitrogen, Eugene, OR). To stain nuclei, cells were incubated with DAPI (Molecular Probes Invitrogen, Eugene, OR). Glasses were mounted with mounting media (Dako, Glostrup, Denmark). Cells were photographed using a TE2000-E fluorescence microscope (Nikon, Tokyo, Japan) equipped with a DXM1200C camera (Nikon).

#### Tissue staining

Tissue from adjacent locations as where pieces for cell isolations were taken was fixed in 4% paraformaldehyde and embedded in paraffin. From each block, sections 5 μm in thickness were generated. Sections were deparaffinized, rehydrated and pre-treated to make epitopes accessible for antibodies. Endogenous peroxidase activity was blocked in 3% hydrogen peroxidase (Merck, Damstadt, Germany) followed by a 30 minutes block with 2% BSA-TBS containing 5% serum raised in the same species as the secondary antibodies used. Furthermore, endogenous avidin and biotin binding sites were blocked (Vector avidin/biotin blocking kit, Vector laboratories, Burlingame, CA) according to the manufacturer’s protocol. Sections were incubated with primary antibodies: rabbit polyclonal antibody against ROCK1 (Abcam, Cambridge, UK), mouse monoclonal antibody against Vimentin (Santa Cruz Biotechnology, Santa Cruz, CA) and mouse monoclonal antibody against prolyl-4 hydroxylase (Acris antibodies, Hiddenhausen, Germany). This was followed by incubation with secondary antibodies:biotin-conjugated goat anti-rabbit (Vector laboratories, Burlingame, CA), biotin-conjugated horse anti-mouse (Vector laboratories, Burlingame, CA). Sections were incubated with avidin and biotin (Vector laboratories, Burlingame, CA) according to the manufacturer’s instructions and were developed with DAB (Vector laboratories, Burlingame, CA) to visualize bound antibodies and then counterstained with Mayer’s hematoxylin. Alternatively, sections were double-stained with primary antibodies as mentioned above and then incubated with Alexa-flour 555 and 647 conjugated secondary antibodies (Molecular Probes Invitrogen, Eugene, OR). To stain nuclei, cells were incubated with DAPI (Molecular Probes Invitrogen, Eugene, OR). Sections were photographed using a TE2000-E fluorescence microscope (Nikon, Tokyo, Japan) equipped with a DXM1200C camera (Nikon, Tokyo, Japan).

### Fibroblast contraction assay

The gels were prepared using a modified form of a protocol that has previously been described [20]. Briefly, 96-well tissue culture plates (Cellstar, Monroe, NC) were coated with 1% BSA overnight and were then washed with PBS. Neutral collagen solution was prepared by mixing 2x DMEM, supplemented with 4% FCS and 10% Glutamine, Collagen type-I solution (PureCol, Inamed Biomaterials, Fremont, CA) and 0.2 M HEPES buffer, pH 8.0 in the volume relation 5:4:1. Fibroblasts, suspended in DMEM (1 × 10^6^ cells/ml) were added to collagen solution in the relation 1:9 (v/v). The final cell density was thus 1 × 10^5^/ml and the final concentration of collagen in the contraction gels was 1.1 mg/ml in DMEM with a physiological ionic strength of 1 × DMEM (pH 7.4) containing 0.4% FCS and 1% Glutamine. 100 μl cell/collagen solution were added to each well and the collagen gels were polymerized for 1 hour at 37°C. After polymerization 100 μl of DMEM supplemented with 0.4% FCS and 1% Glutamine was gently added to each well. Gels were released with a spatula 4 hour after polymerization and were photographed using a camera (Sony, Tokyo, Japan). The gel area at this point was used as the initial area. The gel area was then monitored over time and was compared to the initial area. All gel contraction experiments are the mean of triplicate measurements. In indicated cases Y27563 or blebbistatin was added to cell suspensions just before they were mixed with the collagen solution.

### Cytoxicity assay

The cytotoxic effect of ROCK inhibitor Y27632 and the Myosin II inhibitor blebbistatin was assessed by trypan blue exclusion. Cells were incubated for 24 hours in the presence of the inhibitors. Trypan blue (Gibco BRL, Paisley, UK) was added to wells and a minimum of 250 cells were counted in each well and the number of living (non-stained) or dead (blue) cells were recorded. A minimum of 4 wells were used for each concentration of the inhibitors.

### Statistics

Data are expressed as mean ± SEM. The Mann–Whitney test was used to compare statistical differences between two groups. The Wilcoxon signed rank test was used to perform a paired comparison of the effect of the inhibitors. Comparison of the inhibitory effect of Y27632 and blebbistatin on the two cell types was done by comparing the fold change with/without the inhibitors. The ratios were then compared with the Mann–Whitney test. The comparison between the two inhibitors was calculated as (Y27632 - untreated) / (blebbistatin - untreated) for the individual cell types and the groups were then compared with the Mann–Whitney test. In some cases it was not possible to establish cell cultures because there was no outgrowth of fibroblasts. In other cases there were few cells in a culture with low proliferative potential. In these cases there were not cells enough for all experiments and therefore the number of patients or controls in each experiment varies. However, no available data have been excluded from the measurements and the statistical analysis. Differences were considered significant at p < 0.05. All analyses were performed using GraphPad Prism software version 4.00 (GraphPad Software, San Diego, CA).

## Results

### Clinical and demographic features

Characteristics of included COPD patients (n = 9) and control subjects (n = 12) are shown in Table 1. 3 out of 9 were males the COPD group while 5 out of 12 were males in the control group. Predicted FEV_1_ was 19.9% in COPD patients and 102.6% for control subjects. All the COPD patients were ex-smokers with a heavy smoking burden whereas 11 of the controls were never smokers and one was an ex-smoker with 8 pack years.

**Table 1:** COPD patients and control subjects in the study

**Characteristics**	**Controls**		**COPD**	
No.	8 + 4*		9	
Age (range)	29*	(23–41)	62	(53–66)
Pack years (range)	1*	(0–8)	39	(25–60)
Gender, M/F in %	42/58		33/67	
**Lung function**				
FEV_1_	4.1*	(3.2–5.4)	0.55	(0.4–0.9)
FEV_1_ % predicted	102.6*	(84–116)	19.3	(14–24)
FVC	5.0*	(4.0–6.4)	2.0	(1.3–2.8)
FEV_1_ % predicted/FVC	21*	(17–25)	30	(20–39)
DLco	m		1.4	(1.4–1.5)^†^
DLco % predicted	m		24	(14–42)^§^

*Values are only from 8 of the control subjects. The other 4 subjects were lung donors with no former history of lung disease. Three out of four had the age interval between 46 and 65 years old and one between 31–45 years old. One of them was a former smoker with a smoking history of 7.5 pack years. There was no other lung function characteristics available for these individuals.

^†^ Only values from 5 patients. ^§^ Only values from 6 patients. m denotes that data is missing. Data is presented as mean (range).

### Phenotypes of centrally and distally derived fibroblasts

**Figure 1:**
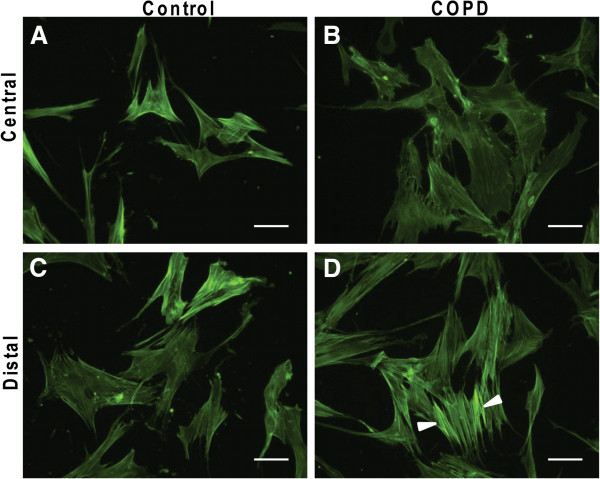
**Stress fiber staining in isolated fibroblasts.** Stress fibers were visualized in centrally (**A** and **B**) and distally (**C** and **D**) derived fibroblasts from control subjects (**A** and **C**) and COPD patients (**B** and **D**) by staining with phalloidin that binds to F-actin. Contractile cells have parallel fibers attached to their lamellipodia as is indicated by arrowheads in (**D**) Other cell types do not have this feature to the same extent. Scale bars represent 50 μm.

**Figure 2:**
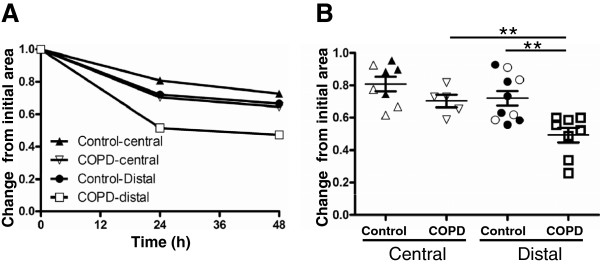
**Collagen gel contraction.** (**A**) The contractile potential of the isolated fibroblasts was monitored in collagen contraction gels over time by comparing gel areas of each time point by the initial gel areas. Each point represents the mean of each study group. (**B**) Contractile potential of isolated fibroblasts after 24 hours compared to the initial area. Open symbols represents fibroblasts isolated from lung explants and closed symbols fibroblasts isolated from lung biopsies. **P < 0.01.

Contractile properties were evaluated in centrally and distally derived fibroblasts from control subjects and COPD patients. Stress fibers were visualized by phalloidin staining as shown in Figure 1A-D. Distally derived fibroblasts from COPD patients had a phalloidin staining pattern with parallel fibers attached to their lamellipodia typical for contractile cells. This was contrasted with the staining pattern in distally derived fibroblasts from control subjects and centrally derived fibroblasts from COPD patients and control subjects. To further evaluate the contractile properties of the cells the ability to contract three-dimensional collagen gels was assessed. Distally derived fibroblasts from COPD patients contracted the gels significantly more than the other cell types at all investigated time points (Figure 2A). After 24 hours distally derived fibroblasts had contracted the gels significantly more (0.49 ± 0.05) than centrally derived fibroblasts from COPD patients (0.70 ± 0.04) (p < 0.01) and also against distally derived fibroblasts from control subjects (0.72 ± 0.01) (p < 0.05), (Figure 2B).

### Expression of proteins involved in fibroblast contraction

**Figure 3:**
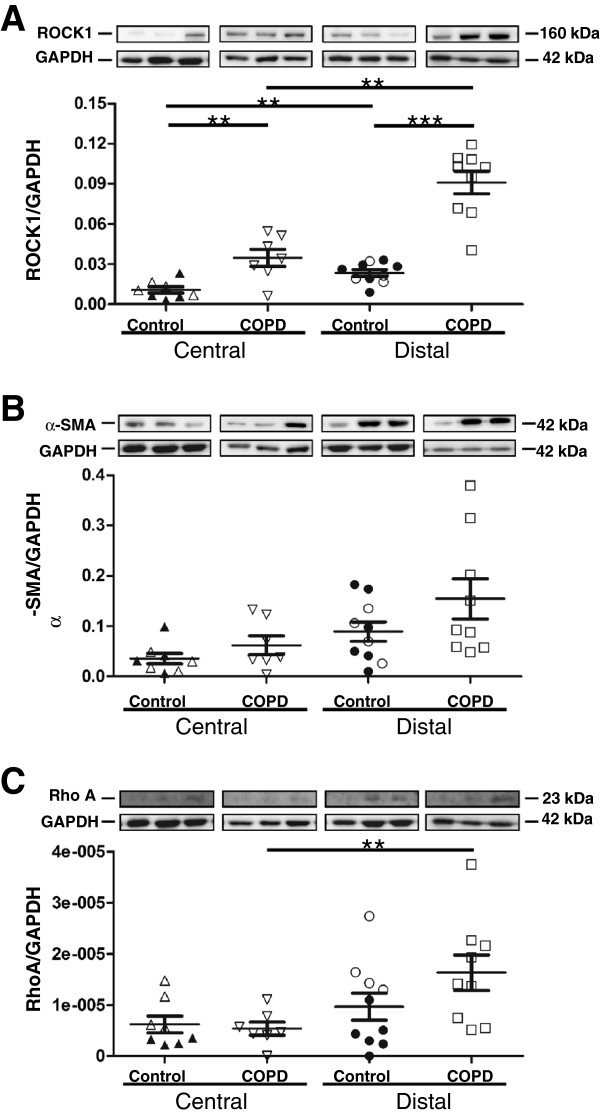
**Expression of proteins involved in fibroblast contraction.** Cell extracts from centrally and distally derived fibroblasts from control subjects and COPD patients were immunoblotted using antibodies against ROCK1 (**A**), α-SMA (**B**), RhoA (**C**), and GAPDH. Blotted bands were quantified with morphometry and values are presented as the intensity of each band relative to the intensity of the loading control: GAPDH. Photos show representative bands. **P < 0.01 and *** < 0.001.

**Figure 4:**
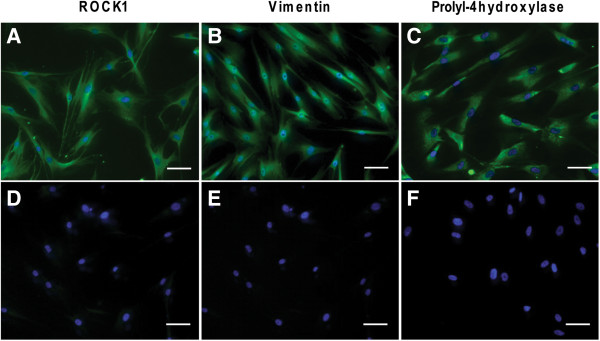
**Immunostaining of fibroblasts from COPD patients.** Isolated fibroblasts from control subjects and COPD patients were immunostained to verify their expression of ROCK1 (**A**) and the mesenchymal markers Vimentin (**B**) and Prolyl-4 hydroxylase (**C**). The control stainings in the absence of primary antibodies for ROCK1 are shown in (**D**), for vimentin in (**E**) and for prolyl-4 hydroxylase in (**F**). Scale bars represent 50 μm.

We next examined the expression of proteins known to be involved in fibroblast contraction: ROCK1, α-SMA and Rho A to elucidate the molecular mechanism for the increased contractility (Figure 3 A-C). Distally derived fibroblasts from COPD patients had significantly higher ROCK1 expression (0.09 ± 0.008) than distally derived fibroblasts from control subjects (0.02 ± 0.002) (p <0.001). Centrally derived fibroblasts from also had significantly higher ROCK1 expression (0.03 ± 0.006) than centrally derived fibroblasts from control subjects (0.01 ± 0.002) (p <0.01). In addition, ROCK1 expression in distally derived fibroblasts from both COPD patients and control subjects were significantly higher than their respective centrally derived counter-parts (p < 0.01 for both comparisons). There was a trend to increased α-SMA expression in distally derived fibroblasts from control subjects compared to centrally derived fibroblasts (p < 0.054) and a similar trend was observed for distally and centrally derived fibroblasts from COPD patients (p < 0.055). Distally derived fibroblasts from COPD patients had significantly higher Rho A expression (1.63 × 10^-5^ ± 3.45 × 10^-6^) compared to centrally derived fibroblasts from COPD patients (5.41 × 10^-6^ ± 1.30 × 10^-6^) (p < 0.05). The cellular expression of ROCK1 in distally derived fibroblast from COPD patients was confirmed by immunohistochemistry as shown in Figure 4A. Moreover, the mesenchymal identity of the fibroblasts was verified by using antibodies against vimentin, a member of intermediate filaments in mesenchymal cells and prolyl-4 hydroxylase, an enzyme involved in collagen synthesis (Figure 4 B-C).

### Role of ROCK and myosin II on fibroblast contraction

**Figure 5:**
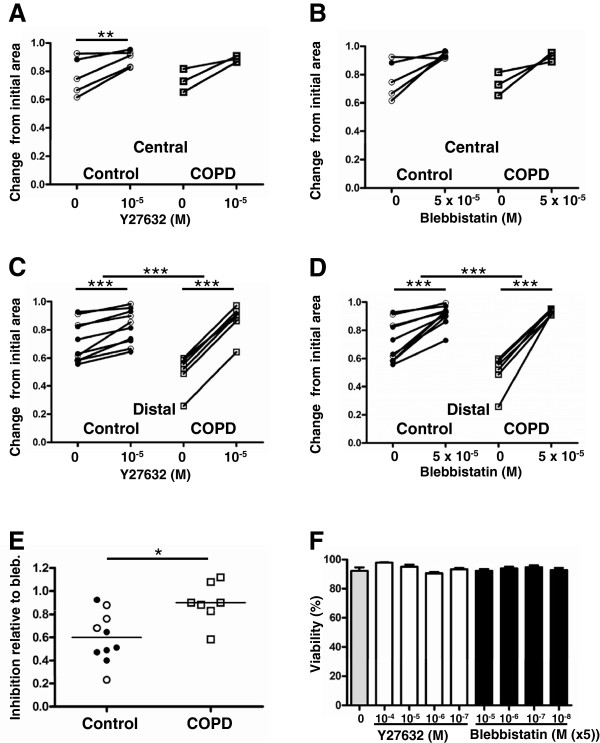
**The effect of ROCK on fibroblast contraction.** The influence of ROCK1 on the contractile potential of isolated fibroblasts was investigated using collagen contraction gels. Cells were pre-treated with different concentration of the ROCK inhibitor Y27632 or the myosin II inhibitor blebbistatin for 1 hour and contraction was monitored as the gel areas after 24 hours compared to the initial gel areas. Paired comparison of centrally derived fibroblasts (**A**) or distally derived fibroblasts (**C**) from control subjects and COPD patients with and without Y27632 after 24 hours incubation. (**B**) paired comparison of centrally derived fibroblasts or distally derived fibroblasts (**D**) from control subjects and COPD patients with and without the myosin II inhibitor:blebbistatin after 24 hours incubation. (**E**) Comparison of the inhibitory effects of Y27632 and blebbistatin calculated as (Y27632-untreated) / (blebbistatin-untreated) after 24 hours incubation for distally derived fibroblasts. (**F**) Cytotoxicity of the used antagonists shown as viability as determined by trypan blue exclusion after 24 hours exposure. Open symbols represents fibroblasts isolated from lung explants and closed symbols fibroblasts isolated from lung biopsies. *P < 0.05, **P < 0.01, *** < 0.001.

We next investigated the contribution of ROCK1 to contraction by using the selective ROCK inhibitor Y27632(Table 2). The concentrations that were used had no effect on cell viability as shown in Figure 5 F. A dose-dependent response was recorded for centrally derived fibroblasts from control subjects (p < 0.05) and for distally derived fibroblasts from COPD patients (p < 0.01) (see Table 2). The inhibitor had less effect on distally derived fibroblasts from control subjects and centrally derived fibroblasts from COPD patients. However, the 10 μM dose significantly inhibited contraction compared to untreated cells in centrally derived fibroblasts from control subjects (p < 0.01) and distally derived fibroblasts from both control subjects and COPD patients (p < 0.001 for both) when the analysis was paired as shown in Figure 5A and C. The inhibitory effect defined as the fold change with and without addition was greater in fibroblasts from COPD patients (p < 0.001) than in fibroblasts from control subjects The myosin II inhibitor, blebbistatin, dose-dependently inhibited contraction in centrally derived fibroblasts from control subjects (p < 0.05) and distally derived fibroblasts from both control subjects (p <0.01) and COPD patients (p < 0.001) (see Table 2 and Figure 5 B and D). The inhibitory effect of Y27632 (10 μM) was next compared to the effect of blebbistatin (50 μM). After 24 hour of incubation, the inhibitory effect of Y27632 (10 μM) compared to blebbistatin (50 μM) was significantly greater in fibroblasts from COPD patients (0.90 ± 0.07) than from control subjects (0.60 ± 0.07) (p < 0.05) (Figure 5 E). This result suggests that contraction was to a higher extent dependent on the activity of ROCK1 in distally derived fibroblasts from COPD patients compared to from control subjects.

**Table 2:** Dose–response of Y27632 and blebbistatin

			**Y27632**			**blebbistatin**	
		**Untreated**^†^	**10 μM**^†^	**1 μM**^†^	**0.1 μM**^†^	**50 μM**^†^	**5 μM**^†^
**Central**	Control (n = 6)	0,77	0,89 *	0,79	0,68	0,94 *	0,76
	COPD (n = 3)	0,73	0,89	0,84	0,80	0,93	0,85
**Distal**	Control(n = 6)	0,72	0,82	0,70	0,70	0,91 **	0,71
	COPD (n = 7)	0,51	0,87 **	0,55	0,59	0,93 ***	0,63

^†^Values show gel contraction after 24 hours compare to initial area at 0 hours.

* p < 0.05 compared to untreated cells, ** p < 0.01 compared to untreated cells, *** p < 0.0001 compared to untreated cells.

### *In vivo* expression of ROCK1 in fibroblasts

**Figure 6:**
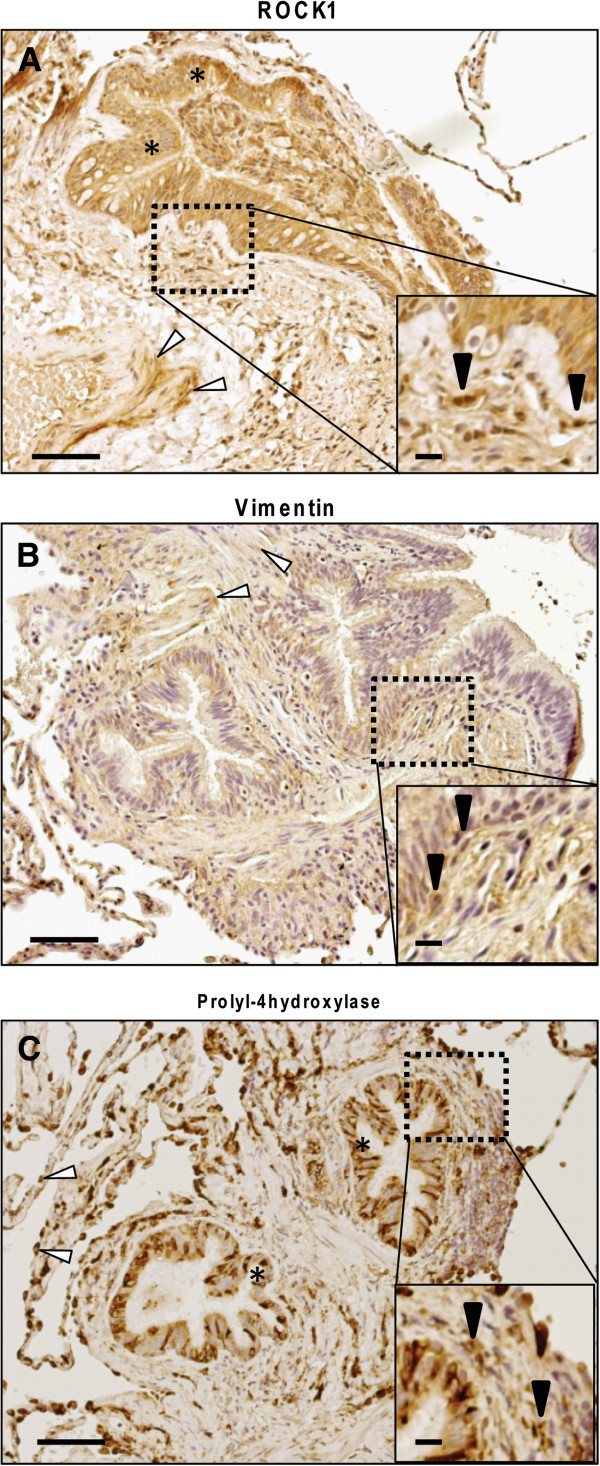
**Fibroblast and ROCK1 staining in lung sections from COPD patients.** Representative micrograph from one COPD patient that show immunostaining with antibodies against ROCK1, vimentin and prolyl-4 hydroxylase. Antibodies were visualized by DAB (shown in brown) and sections were counterstained with Mayer’s hematoxylin, which stains cell nuclei blue. (**A**) showsROCK1 staining. Asterisks indicate the bronchiolar epithelium. Smooth muscle cells are indicated by open arrowheads. (**B**) shows vimentin staining. Open arrowhead show smooth muscle cells. (**C**) shows prolyl-4 hydroxylase staining. Asterisks show immuno-positive bronchiolar epithelial cells. Alveolar type II cells are indicated by open arrowheads. Inserts show subepithelial, elongated, spindle-like immuno-positive cells (indicated by closed arrowheads). Scale bars represent 50 μm of the larger image and 10 μm on inserts.

**Figure 7:**
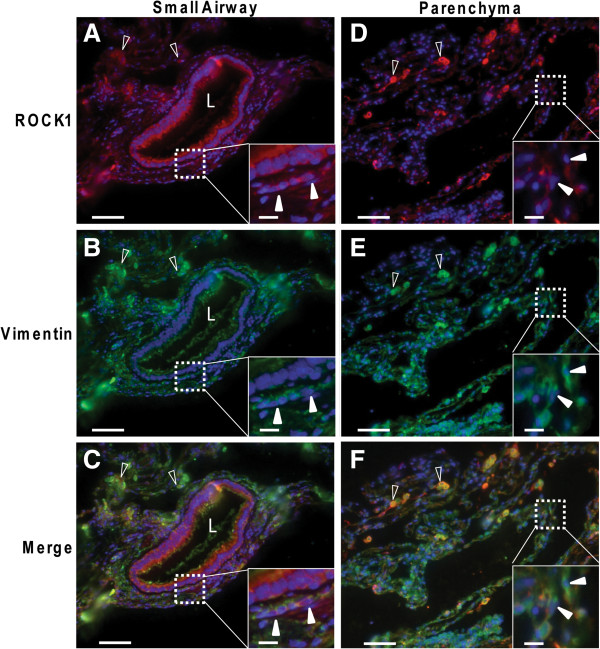
**Double staining of vimentin and ROCK1.** Immunostaining for ROCK1 (**A** and **D**) and vimentin (**B** and **E**) in COPD tissue sections. Cell nuclei are visualized by DAPI staining, shown in blue. (**C**) and (**F**) show merged images. (**A**), (**B**) and (**C**) show a small airway and (**D**), (**E**) and (**F**) alveolar parenchyma. Inserts show cells positive for both ROCK1 and vimentin with fibroblast-like morphology as indicated by closed arrowheads. Open arrowheads show rounded double-positive cells that are likely to be alveolar macrophages. Scale bars represent 50 μm of the larger image and 10 μm on inserts.

Immunohistochemistry was used to identify the presence of ROCK1-positive fibroblasts in tissue sections from COPD patients. Since there are few markers that are exclusive for fibroblasts we first evaluated the immunostaining of antibodies against ROCK1, vimentin and prolyl-4 hydroxylase in the submucosa of bronchioles, a location where fibroblasts normally can be found. Immunoreactivity for ROCK1 was identified in bronchiolar epithelial cells, in smooth muscle cells and, in addition, in elongated, spindle-shaped cells located in the lamina propria that is likely to be fibroblasts (Figure 6 A). Immunoreactivity for vimentin was mainly identified in smooth muscle cells and in subepithelial fibroblast-like cells (Figure 6 B). Prolyl-4 hydroxylase immunoreactivity was identified in bronchiolar epithelial cells, in globular alveolar cells that are likely to be type II pneumocytes as has previously described [21] and in subepithelial fibroblast-like (Figure 6 C). As shown in Figure 7 A-C there were elongated, fibroblast-like cells double-positive for vimentin and ROCK1 in the bronchiolar submucosa. Figure 7 D-F show that double-positive cells also could be found in the alveolar parenchyma. Additionally, large rounded cells with a punctuated staining pattern for the two antibodies were identified in the alveolar parenchyma (Figure 7 C and F). These cells are likely to be alveolar macrophages.

## Discussion

In this study we show that distally derived fibroblasts from patients with severe COPD are more contractile than fibroblasts from control subjects. The enhanced contractility is dependent on ROCK1 expression and function, as cells from COPD patients have a higher expression of ROCK1 and contraction was inhibited by the ROCK1 inhibitor Y27632. Finally, staining of tissue sections from COPD patients showed the presence of ROCK1 expressing fibroblasts-like cells in small airways and in alveolar parenchyma which suggests that the observed alterations may be relevant *in vivo.* We suggest that, in the COPD lung, factors in the deteriorating environment trigger differentiation of fibroblasts into a contractile phenotype. This alteration may affect the elastic dynamics of small airways and the parenchyma in late stages of COPD.

ROCK1 has been shown to be a central player in the formation of stress fibers [22,23]. It mediates this via multiple mechanisms which result in phosphorylation of myosin light chain [24-26]. Several studies have suggested the Rho/ROCK pathway to be involved in the cellular response to increased matrix stiffness by promoting myofibroblast differentiation and the formation of stress fibers [27,28]. In an elegant study Liu *et. al.* showed that increasing matrix stiffness induced the transition from a quiescent fibroblast phenotype into an active myofibroblast-like phenotype [13]. The transition was suggested to be regulated by the relative balance of the expression of COX-2/prostaglandin E_2_ and Rho/ROCK. However, emphysema is characterized by hyperinflation and degradation rather than fibrotic deposition of the extracellular matrix as is the case in fibrotic pulmonary diseases such as cystic fibrosis and idiopathic pulmonary fibrosis. In addition, the inflammatory process is different in these diseases compared to COPD, which may suggest that there are different mechanisms that drive the phenotypic transition of fibroblasts into a contractile phenotype [29-31].

In conflict with our data, Togo et al. reported that fibroblasts from moderate to severe COPD patients have reduced repair capabilities defined as an attenuated potential to contract and migrate [32]. The authors partially explained the altered phenotype by enhanced expression of COX-2/PGE2 and partially by unresponsiveness to TGF-β1. We have in a previous study used cells isolated from the same donors as in the present study to examine the fibroblast production of different proteoglycans [17]. The data showed that TGF-β1 induced a similar increase in the production of versican, perlecan, and biglycan in fibroblasts from both COPD patients and control subjects, which suggests that the TGF-β1 response in this respect was not affected. One explanation to the opposing data may be differences in the study groups. While Togo *et al.* investigated fibroblasts from patients with moderate to severe COPD (FEV_1_ % ranging from 19–67 with the mean 44%) all of our patients had very severe COPD (FEV_1_ % ranging from 14–24 with the mean 20%). The deviating data may thus indicate that different fibroblast repair mechanisms are activated in lungs from different disease stages.

The differentiation of fibroblasts into myofibroblasts is accompanied by enhanced expression of α-SMA which is incorporated into stress fibers [14]. Stress fibers that contain α-SMA generate more contractile force than normal stress fibers that only contain β- and γ-actin [33]. In the present study there was no significant difference in expression of α-SMA between fibroblasts from COPD patients and control subjects although the contractile capability differed. However, contractile force is generated both by α-SMA and the Rho/ROCK pathway, and the two cell types had similar expression of α-SMA but fibroblasts from COPD patients had higher expression of ROCK1 which may explain the difference.

Recently, it was suggested that there are unique fibroblast populations in central airways and in the lung parenchyma [15,17,18]. Distally derived fibroblasts have been shown to have higher α-SMA expression than centrally derived fibroblasts [16]. In the present study there was a trend that distally derived fibroblasts had higher expression of α-SMA than centrally derived fibroblasts both from control subjects and COPD patients. However we recorded a difference in the expression of ROCK1 between centrally and distally derived fibroblasts both from control subjects. In addition, in severe COPD patients we could extend this comparison to also include contractile potential.

In the current study we identified fibroblast-like cells that were immuno-positive for fibroblast markers and ROCK1 in small airways and in the alveolar parenchyma. These data suggests that the present functional *in vitro* data also may be relevant *in vivo*. The altered phenotype may be a compensatory mechanism to the loss of elastic fibers and specific extracellular matrix molecules, as has been reported in the parenchyma of COPD patients [34].

The primary fibroblasts used in the present study came from two sources. Fibroblasts from COPD patients were isolated from lung explants while control fibroblasts were obtained from both biopsies and from lung explants from control subjects. When isolating distal fibroblasts from explants there is always a risk of contamination with small airways although care was taken to avoid it. However, we could not see any skewness due to the different isolation sources in the assays we used with exception for the RhoA western blot where the controls were stratified due to origin of fibroblasts. Furthermore, it has been shown that glucocorticoids may enhance fibroblast gel contraction. All COPD patients in this study were taking glucocorticoids on regular basis and we can therefore not exclude that some of the investigated parameters were affected by these drugs [35]. The two groups were poorly age-matched, and it cannot be excluded that some of the observed results were a result of these differences. However, to account for the poor age-match between the study groups, fibroblasts from donor lungs (from control subjects similar in age as the COPD patients) were also included and these cells did not differ from the other control cells in the assays used. The COPD patients in the present study were all ex-smokers while the controls were non-smokers (except for one subject). As have been shown by Wang *et al.* cigarette smoke may induce contraction of high-density primary fibroblasts cultures and it can therefore not be ruled out that some of the observed changes in the present study are a result of chronic inflammation induced by smoking [36].

## Conclusions

In summary, in this study we report that fibroblasts isolated from the parenchyma from patients with severe COPD have a more contractile phenotype. This altered phenotype was dependent on ROCK1, as ROCK1 expression was found to be increased and the selective ROCK-inhibitor Y27632 blocked contraction. This alteration may be important for the elastic dynamic in severe stages of COPD.

## Abbreviations

COPD: Chronic obstructive pulmonary disease; ROCK1: Rho-associated coiled-coil protein kinase 1; α-SMA: α-smooth muscle actin; COX-2: Cyclooxygenase-2; PGE2: Prostaglandin E2; FEV1: Forced expiratory volume in 1 second; MLCK: Myosin light chain kinase.

## Competing interests

**OH, SR, AAR, KN, JSB** and **GWT** declare that they have no competing interests.

**EW, MKR, MD, LE** is employees or former employees of AstraZeneca R&D.

**LB** has received payments lecture fees from the following companies: AZ, Airsonett, Boehringer Ingelheim, Novaartis GlaxoSmithKline, Niigard, Merck, Mundipharma, Pfizer and UCB and have been involved in the adivisory board activities for AZ, Airsonett, Boehringer Ingelheim, Novaartis GlaxoSmithKline, Niigard, Merck, Pfizer and UCB. None of these commitments are in conflict with any part of the present study.

**CGL** has received payments for lectures and ad hoc advisory boards from Astrazeneca, Boehringer Ingelheim, Novaartis GlaxoSmithKline, Daxas and Meda, and has received institutional grants from Astrazeneca, Boehringer Ingelhem and GlaxoSmithKline.

## Authors’ contributions

**OH** conceived and designed the experiments, performed western blots, contraction assays and the immunohistochemistry, analyzed the data, drafted the manuscript. **SR** helped to carry out western blots and helped to draft the manuscript, **AAS**, helped out with immunohistochemistry and drafted the manuscript. **KN** isolated fibroblasts. **EW** conceived and designed the experiments and helped to draft the manuscript. **MKR** conceived and designed the experiments and helped to draft the manuscript. **MD** contributed isolating fibroblasts and helped to draft the manuscript. **LE** conceived and designed the experiments and helped to draft the manuscript. **LB** helped to draft the manuscript. **JSE** helped to draft the manuscript. **CGL** conceived and designed the experiments and helped to draft the manuscript. **GWT** conceived and designed the experiments, helped analyzing the data and helped to draft the manuscript. All authors read and approved the final manuscript.

## References

[B1] Barnes PJ Shapiro SD Pauwels RA Chronic obstructive pulmonary disease: molecular and cellular mechanisms Eur Respir J 2003 22 672 688 10.1183/09031936.03.00040703 14582923

[B2] Hogg JC Chu F Utokaparch S Woods R Elliott WM Buzatu L Cherniack RM Rogers RM Sciurba FC Coxson HO Pare PD The nature of small-airway obstruction in chronic obstructive pulmonary disease N Engl J Med 2004 350 2645 2653 10.1056/NEJMoa032158 15215480

[B3] Spurzem JR Rennard SI Pathogenesis of COPD Semin Respir Crit Care Med 2005 26 142 153 10.1055/s-2005-869535 16088433

[B4] Baraldo S Lokar Oliani K Turato G Zuin R Saetta M The Role of Lymphocytes in the Pathogenesis of Asthma and COPD Curr Med Chem 2007 14 2250 2256 10.2174/092986707781696573 17896974

[B5] Gamble E Grootendorst DC Hattotuwa K O'Shaughnessy T Ram FS Qiu Y Zhu J Vignola AM Kroegel C Morell F Airway mucosal inflammation in COPD is similar in smokers and ex-smokers: a pooled analysis Eur Respir J 2007 30 467 471 10.1183/09031936.00013006 17504799

[B6] Serini G Gabbiani G Mechanisms of myofibroblast activity and phenotypic modulation Exp Cell Res 1999 250 273 283 10.1006/excr.1999.4543 10413583

[B7] Zhang K Rekhter MD Gordon D Phan SH Myofibroblasts and their role in lung collagen gene expression during pulmonary fibrosis. A combined immunohistochemical and in situ hybridization study Am J Pathol 1994 145 114 125 7518191 PMC1887314

[B8] Tomasek JJ Gabbiani G Hinz B Chaponnier C Brown RA Myofibroblasts and mechano-regulation of connective tissue remodelling Nat Rev Mol Cell Biol 2002 3 349 363 10.1038/nrm809 11988769

[B9] Desmouliere A Geinoz A Gabbiani F Gabbiani G Transforming growth factor-beta 1 induces alpha-smooth muscle actin expression in granulation tissue myofibroblasts and in quiescent and growing cultured fibroblasts J Cell Biol 1993 122 103 111 10.1083/jcb.122.1.103 8314838 PMC2119614

[B10] Hinz B Gabbiani G Cell-matrix and cell-cell contacts of myofibroblasts: role in connective tissue remodeling Thromb Haemost 2003 90 993 1002 14652629 10.1160/TH03-05-0328

[B11] Pellegrin S Mellor H Actin stress fibres J Cell Sci 2007 120 3491 3499 10.1242/jcs.018473 17928305

[B12] Tomasek JJ Vaughan MB Kropp BP Gabbiani G Martin MD Haaksma CJ Hinz B Contraction of myofibroblasts in granulation tissue is dependent on Rho/Rho kinase/myosin light chain phosphatase activity Wound Repair Regen 2006 14 313 320 10.1111/j.1743-6109.2006.00126.x 16808810

[B13] Liu F Mih JD Shea BS Kho AT Sharif AS Tager AM Tschumperlin DJ Feedback amplification of fibrosis through matrix stiffening and COX-2 suppression J Cell Biol 2010 190 693 706 10.1083/jcb.201004082 20733059 PMC2928007

[B14] Darby I Skalli O Gabbiani G Alpha-smooth muscle actin is transiently expressed by myofibroblasts during experimental wound healing Lab Invest 1990 63 21 29 2197503

[B15] Kotaru C Schoonover KJ Trudeau JB Huynh ML Zhou X Hu H Wenzel SE Regional fibroblast heterogeneity in the lung: implications for remodeling Am J Respir Crit Care Med 2006 173 1208 1215 10.1164/rccm.200508-1218OC 16543551 PMC2662967

[B16] Pechkovsky DV Hackett TL An SS Shaheen F Murray LA Knight DA Human lung parenchyma but not proximal bronchi produces fibroblasts with enhanced TGF-beta signaling and alpha-SMA expression Am J Respir Cell Mol Biol 2010 43 641 651 10.1165/rcmb.2009-0318OC 20061511

[B17] Hallgren O Nihlberg K Dahlback M Bjermer L Eriksson LT Erjefalt JS Lofdahl CG Westergren-Thorsson G Altered fibroblast proteoglycan production in COPD Respir Res 2010 11 55 10.1186/1465-9921-11-55 20459817 PMC2886021

[B18] Nihlberg K Andersson-Sjoland A Tufvesson E Erjefalt JS Bjermer L Westergren-Thorsson G Altered matrix production in the distal airways of individuals with asthma Thorax 2010 65 670 676 10.1136/thx.2009.129320 20685740

[B19] Malmstrom J Larsen K Hansson L Lofdahl CG Norregard-Jensen O Marko-Varga G Westergren-Thorsson G Proteoglycan and proteome profiling of central human pulmonary fibrotic tissue utilizing miniaturized sample preparation: a feasibility study Proteomics 2002 2 394 404 10.1002/1615-9861(200204)2:4<394::AID-PROT394>3.0.CO;2-6 12164698

[B20] Gullberg D Tingstrom A Thuresson AC Olsson L Terracio L Borg TK Rubin K Beta 1 integrin-mediated collagen gel contraction is stimulated by PDGF Exp Cell Res 1990 186 264 272 10.1016/0014-4827(90)90305-T 2298242

[B21] Kasper M Fuller SD Schuh D Muller M Immunohistological detection of the beta subunit of prolyl 4-hydroxylase in rat and mini pig lungs with radiation-induced pulmonary fibrosis Virchows Arch 1994 425 513 519 7850076 10.1007/BF00197555

[B22] Ishizaki T Maekawa M Fujisawa K Okawa K Iwamatsu A Fujita A Watanabe N Saito Y Kakizuka A Morii N Narumiya S The small GTP-binding protein Rho binds to and activates a 160 kDa Ser/Thr protein kinase homologous to myotonic dystrophy kinase EMBO J 1996 15 1885 1893 8617235 PMC450107

[B23] Leung T Chen XQ Manser E Lim L The p160 RhoA-binding kinase ROK alpha is a member of a kinase family and is involved in the reorganization of the cytoskeleton Mol Cell Biol 1996 16 5313 5327 8816443 10.1128/mcb.16.10.5313 PMC231530

[B24] Amano M Ito M Kimura K Fukata Y Chihara K Nakano T Matsuura Y Kaibuchi K Phosphorylation and activation of myosin by Rho-associated kinase (Rho-kinase) J Biol Chem 1996 271 20246 20249 10.1074/jbc.271.34.20246 8702756

[B25] Velasco G Armstrong C Morrice N Frame S Cohen P Phosphorylation of the regulatory subunit of smooth muscle protein phosphatase 1 M at Thr850 induces its dissociation from myosin FEBS Lett 2002 527 101 104 10.1016/S0014-5793(02)03175-7 12220642

[B26] Hagerty L Weitzel DH Chambers J Fortner CN Brush MH Loiselle D Hosoya H Haystead TA ROCK1 phosphorylates and activates zipper-interacting protein kinase J Biol Chem 2007 282 4884 4893 17158456 10.1074/jbc.M609990200

[B27] Goffin JM Pittet P Csucs G Lussi JW Meister JJ Hinz B Focal adhesion size controls tension-dependent recruitment of alpha-smooth muscle actin to stress fibers J Cell Biol 2006 172 259 268 10.1083/jcb.200506179 16401722 PMC2063555

[B28] Wipff PJ Rifkin DB Meister JJ Hinz B Myofibroblast contraction activates latent TGF-beta1 from the extracellular matrix J Cell Biol 2007 179 1311 1323 10.1083/jcb.200704042 18086923 PMC2140013

[B29] Gross TJ Hunninghake GW Idiopathic pulmonary fibrosis N Engl J Med 2001 345 517 525 10.1056/NEJMra003200 11519507

[B30] Jacquot J Tabary O Le Rouzic P Clement A Airway epithelial cell inflammatory signalling in cystic fibrosis Int J Biochem Cell Biol 2008 40 1703 1715 10.1016/j.biocel.2008.02.002 18434235

[B31] Barnes PJ The cytokine network in chronic obstructive pulmonary disease Am J Respir Cell Mol Biol 2009 41 631 638 10.1165/rcmb.2009-0220TR 19717810

[B32] Togo S Holz O Liu X Sugiura H Kamio K Wang X Kawasaki S Ahn Y Fredriksson K Skold CM Lung fibroblast repair functions in patients with chronic obstructive pulmonary disease are altered by multiple mechanisms Am J Respir Crit Care Med 2008 178 248 260 10.1164/rccm.200706-929OC 18467512 PMC2542423

[B33] Hinz B Celetta G Tomasek JJ Gabbiani G Chaponnier C Alpha-smooth muscle actin expression upregulates fibroblast contractile activity Mol Biol Cell 2001 12 2730 2741 11553712 10.1091/mbc.12.9.2730 PMC59708

[B34] Black PN Ching PS Beaumont B Ranasinghe S Taylor G Merrilees MJ Changes in elastic fibres in the small airways and alveoli in COPD Eur Respir J 2008 31 998 1004 10.1183/09031936.00017207 18216063

[B35] Wen FQ Skold CM Liu XD Ertl RF Zhu YK Kohyama T Wang H Rennard SI Glucocorticoids and TGF-beta1 synergize in augmenting fibroblast mediated contraction of collagen gels Inflammation 2001 25 109 117 10.1023/A:1007170622699 11321357

[B36] Wang H Liu X Umino T Kohyama T Zhu YK Wen FQ Spurzem JR Romberger DJ Kim HJ Rennard SI Effect of cigarette smoke on fibroblast-mediated gel contraction is dependent on cell density Am J Physiol Lung Cell Mol Physiol 2003 284 L205 213 12388358 10.1152/ajplung.00042.2002

